# John Ewens, L.R.C.P. Lond., L.R.C.S. Edin.

**Published:** 1916-12

**Authors:** 


### Obituary.

---

### JOHN EWENS, L.R.C.P. Lond., L.R.C.S. Edin.

With much regret we have to record the death of one who represented our medical confrères of the last century, for Mr. John Ewens was in his eighty-seventh year, when, after a few days' illness, he passed quietly away on December 14th.

He passed his student career at St. George's Hospital, London, qualifying in 1851. From 1876 to 1896 he was Honorary Surgeon to the Royal Hospital for Sick Children and Women, and subsequently Honorary Consulting Surgeon, as well as Consulting Surgeon to the Bristol Home for Blind Women.

But though a familiar figure in medical practice, and at the meetings of the Medico-Chirurgical Society so many years ago, he was a remarkably active man till within the last few years, and was visiting his patients within a month of his death. No one whose privilege it was to know Mr. Ewens can have failed to be impressed with his kindly disposition and great courtesy. He was a keen Churchman, and known as a strong upholder of evangelical views. Though reaching a ripe old age, he still had a very large circle of friends, not confined to his own day and generation, which indeed he had largely outlived, but amongst his juniors, by whom he was much beloved.

